# Predictors of caregiver burden in caregivers of older people with physical disabilities in a rural community

**DOI:** 10.1371/journal.pone.0277177

**Published:** 2022-11-04

**Authors:** Denis Tuttle, Jiranan Griffiths, Anuchart Kaunnil

**Affiliations:** 1 Department of Rehabilitation Science, University at Buffalo, Buffalo, New York, United States of America; 2 Department of Occupational Therapy, Faculty of Associated Medical Sciences, Chiang Mai University, Chiang Mai, Thailand; Chiang Mai University, THAILAND

## Abstract

Caring for an aging society is a problem facing many countries including Thailand. This cross-sectional study investigated caregiver burden and related predictive factors among 69 caregivers who had older family members with physical disabilities. Burden Scale, World Health Organization Quality of Life-Bref Thai (QOL), Patient Health Questionnaire-9 (PHQ), Barthel Activity of Daily Living Index (ADL), and Lawton-Brody Instrumental Activities of Daily Living Scale (IADL) assessments were used in addition to demographic data. Thirteen caregivers (18.8%) reported no caregiver burden, 30 (43.5%) reported low-moderate burden, 21 (30.4%) reported moderate-high burden and 5 (7.2%) reported high burden. Using Fisher’s Exact Test the factors found to be significantly associated to caregiver burden were: categorical age of the caregiver (p = .000), education level of the caregiver (p = .002), relationship to the care recipient (p = .009), categorical income level of the caregiver (p = .041), QOL of the caregiver (p = .001) and ADL status of the care recipient (p = .003). Forward stepwise linear regression model revealed three factors which were PHQ score (β = .543, p < .000), ADL score (β = -.341, p = .001) and hours of care/week (β = .227, p = .017). Future studies should focus on interventions that impact depression levels, independence with activities of living and hours of care per week.

## Introduction

Globally, the population aged 60 years or older was 1 billion in 2020 [1], this has more than doubled since 1980 when it was approximately 382 million [2]. It is expected to double by 2050 with a projected number of 2 billion. Developing regions have two thirds of the world’s older persons and that is expected to increase to nearly 80% by 2050 [1]. In 2021, it was reported that Thailand had 18% of the population being 60 years or older, with the expectation that in 2022 it will meet the classification as a “complete aged society” society, where 21% of the population is 60 years or older [3–7].

As of 2018, there were 616,365 older adults with physical disabilities in Thailand [8]. Physical disabilities are defined as, “a condition of the body that makes it more difficult for the person with the condition to do certain activities and interact with the world around them.” [9]. Thailand has an established culture of families providing assistance for their older parents and relatives [10]. Children, spouses and relatives feel a duty to care for older family members [11, 12] Women most typically take this role caring for spouses, parents, parents-in-law and children [13]. Ninety percent of both older adults and those with disabilities receive daily familial care [13]. This leaves the significant responsibility of caregiving on family members [11]. Having an increasingly aging population, Thailand will see an increase in familial caregiving in the coming years [14].

The increasing average age and related increasing number of older people with disabilities is complicated by several factors reducing the number of caregivers. A reduction in the average number of children per family from 7 in 1974 to 1.5 in 2000 leaves fewer potential family caregivers [7]. Younger people are moving from rural areas to more urban environments leaving fewer family members to care for older people [13]. The changes to the family structure and geographic changes of families when paired with an increasing aging population will increase the burden on caregivers [15]. Caregiver burden is the physical, financial and psychosocial hardships of caring for a loved one who struggles with a medical condition [12, 16]. Caregiver burden is associated with higher depression levels and lower quality of life of caregivers [17, 18]. Other studies that have researched caregiver burden in those that care for people with physical disabilities have found that the independence level of the care recipient in activities of daily living and long care giving hours are associated factors [15, 19]. Family caregivers play an important role in providing care for the person with a disability who may not be independent to complete their daily activities. They are not paid for providing care and they are also likely to have less time for themselves for sleeping, leisure, and to earn an income. This experience may cause emotional stress, poor health, and a poor quality of life. A study reported that caregivers are not the priority to focus on in the public health system [20]. The Thai government’s policies focus on aging care in terms of health services and developing the quality of life of older people. Therefore it is important to focus on family caregivers to investigate the burden to support better care on aging [5, 21].

Ontai is a sub-district in a rural area in San Kamphaeng district, Chiang Mai province. Most of the residents are farmers and most of the older people have a low education [22]. Ontai has a high percentage of older people living in the community at 26% in 2021. This percentage in the community almost reaches the criterion of a super aging society of 28% [6]. Of that 26%, there were 100 7% registered as having a physical disability [23]. Ontai is a typical rural village where the health care system has attempted to face the issues associated with caring for older people. There is a system where nurses and health volunteers who work in the community health promotion hospital complete home visits for older people with physical disability and their families every month. There is still a known need for support from a multidisciplinary team of health professionals to train and assist caregivers, improving their capability, quality of care and reduce burnout from caring for people with disabilities [24].

There has been substantial research internationally related to caregiver burden experienced by those caring for people with conditions such as dementia and Alzheimer’s disease [11, 22]. There is also increasing research being published regarding caregiver burden for those who care for people with physical disabilities, however, the research on caregivers caring for older people with physical disabilities in rural communities is limited [25, 26]. The cultural differences related to caregiving in an Asian country also warrant specific studies which are lacking. This is especially true of the differences experienced by rural versus urban caregivers [27, 28].

This research investigates:

1. Caregiver factors associated with caregiver burden
2. Level of caregiver burden, depression, quality of life, and correlation with caregiver burden
3. Care recipient factors associated with caregiver burden
4. Predictors of caregiver burden in care providers of older persons with physical disabilities

## Materials and methods

**Fig 1:**
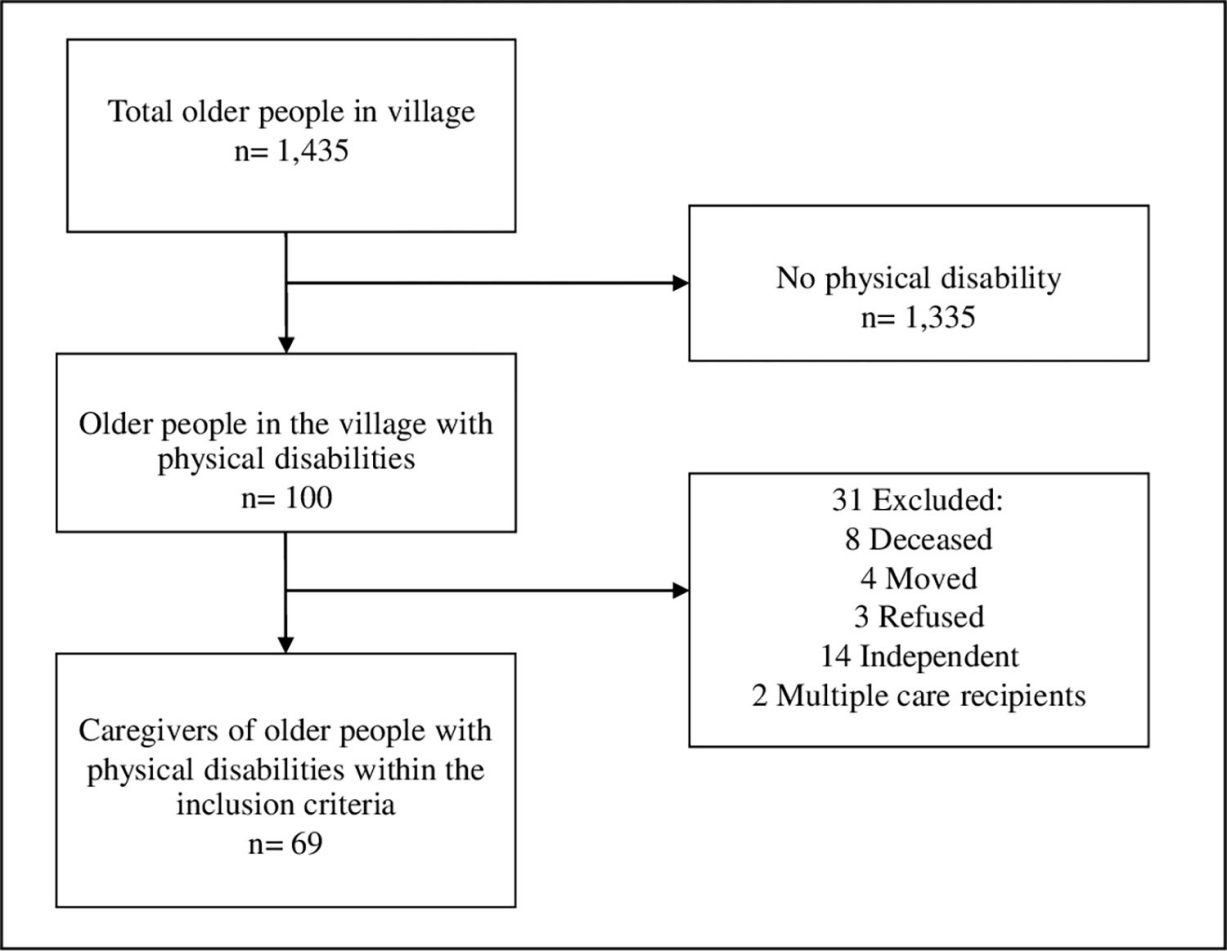
Diagram of the participant selection process.

A cross-sectional design was used in this study. The research was conducted in Ontai, Chiang Mai, Thailand which has 11 smaller villages. Purposive sampling was used for selecting the representative sample of participants that meet the inclusion criteria. According to G power (3.1.9.4) for multiple linear regression, the minimum sample size needed was calculated to be 77 (α = 0.05 and power = 0.8, and medium effect size, predictors = 3) [29]. At the beginning the municipal office records show that there were 1,435 older people registered. Of the 1,435, 100 are also registered as having a physical disability and were recruited for the study. Thirty-one were excluded, eight were deceased, four had moved, three refused to participate, 14 were independent and did not have a caregiver. There were two instances of 1 caregiver providing care for 2 older people with a disability in the same household. To avoid duplicate caregiver information, only one care recipient data was included along with the caregiver data. This resulted in 69 total caregivers available to be participants (Fig 1).

Inclusion criteria for the caregivers were as follows: 1) a willingness to participate, 2) aged 18 years old or older, 3) being able to communicate, 4) being the primary caregiver for a minimum of 1 year, 5) not being paid for the care provided, 6) having a familial relationship with the older patient and 7) providing care to someone over 60 years old with a physical disability.

Occupational therapists were trained on how to conduct the assessments. The training of the occupational therapists consisted of going through each of the assessments with a researcher. Health volunteers along with occupational therapists visited the caregivers or the care recipient’s residence to complete the assessments with the caregiver. The assessments were carried out by occupational therapists (OT) in person, who asked questions of the caregiver. The caregivers were provided answer choices from the questionnaires and the OT recorded the results. As needed, the questionnaire answer options were repeated for the caregiver.

The assessments were conducted in February 2022 and followed all COVID protocols. Masks were worn, assessments were completed outside where possible and hand sanitizer was made available. An authoring researcher was located at the community hospital and was available by phone if any questions arose during the assessment process.

### Instruments

Patient Health Questionnaire-9 (PHQ-9), Burden Scale (BS), World Health Organization Quality of Life-Bref Thai (QOL), Barthel Activity of Daily Living Index (ADL), Lawton-Brody Instrumental Activities of Daily Living Scale (IADL), and demographic data were collected. All English based assessments used have previously been translated to Thai and adjusted for cultural contexts in previous publications with adequate psychometric properties [30–34].

The Patient Health Questionnaire-9 (PHQ-9) was used to assess depression. The 9 questions range from 0 (not at all) to 3 (nearly every day), Likert scale assessment shows the severity of depression. A score of 10 or greater was used to indicate major depression. Validation of the Thai version of the PHQ-9 found satisfactory internal consistency (Cronbach’s alpha at 0.79). As a continuous measure the sensitivity and specificity were 0.84 and 0.77 respectively [30].

The Burden Scale was used to assess caregiver burden. There are 12 questions on a 1–5 Likert scale, 1 (never) to 5 (always). A score of less than 17 is ‘no to trivial burden’, 17–28 ‘low to moderate burden’, 29–40 ‘moderate to high burden’, more than 40 is ‘high burden’. A study found the internal consistency to be 0.86 when using Cronbach’s Alpha. This was tested in 137 family caregivers of older persons [33].

The World Health Organization Quality of Life-Bref Thai (WHOQOL-BREF Thai) was used to assess quality of life. This 26 item, 1–5 Likert scale assessment looks at quality of life by asking questions related to physical, psychological, social relationships and environments. A study found that the reliability of the WHOQOL-Bref Thai with Cronbach’s alpha coefficient of 0.84 and content validity of 0.65 when compared to the WHOQOL Thai [32].

The Thai version of the Barthel Index for Activities of Daily Living (TVBI) was used to assess the degree of independence with activities of daily living (ADL) of the care recipient as reported by the caregiver. This 10 item assessment relates to the care recipient’s ability to complete feeding, bathing, grooming, dressing, bowel control, bladder control, toileting, transfers, mobility and stairs either independently or with a level of assistance. A score of 0–4 indicates being bed bound, 5–11 indicates home bound and a score greater than 12 is social bound. Psychometrics of the TBVI of elderly participants with femur fractures were as follows. All 10 items on the TVBI had good content validity. The reliability of interobserveration was 0.714 and of intraobservation was 0.968. The reliability of the instrument was demonstrated by an acceptable Cronbach’s alpha score of 0.694 [34].

Lawton-Brody Instrumental Activities of Daily Living (IADL) Scale was used to assess Instrumental Activities of Daily Living (IADLs). This 8 item assessment gives a score of 0 or 1 for each item: shopping, laundry, using the telephone, food preparation, housekeeping, financial management, medication management and transportation. A higher total number indicates greater independence with IADLs. A study found the content consistency yielded an acceptable IOC of 0.8–1.0 and presented face validity proper for its measured values in the Thai-version IADL Scale [35].

Demographic data of the caregiver were collected and included, sex, age, marital status, education, employment, income level, relationship to care recipient, if they live with the care recipient and the number of older people in the home. For the care recipients, sex and age were recorded.

### Ethical consideration

Ethical approval for the research was approved by the Ethics Committee, Faculty of Associated Medical Sciences, Chiang Mai University, and study code: AMSEC-64EX-122 number: 21/2022. Data was collected from only the perspective and perception of the caregiver in caring for a family member with a physical disability. There were no observations and recordings of the care recipient during data collection. There was no private information collected of the care recipient. Caregivers were informed of the study objectives and data confidentiality, and written consent was signed by the caregivers with the understanding that they could end their participation in the research at any time. The names and private information of participants were kept confidential.

### Statistical analysis

All statistical analyses were completed using SPSS (IBM Version 26) with a p-value < 0.05 being used for statistical significance. Descriptive statistics including frequency, percentage, mean and standard deviation were used to analyze basic information related to the status of the caregiver and care recipient. Fisher’s Exact Te**s**t was used to determine the association between categorical caregiver burden and caregiver demographic factors. Kolmogorov- Smirnov was used to test the normality of the distribution for caregiver burden scores. The results showed there was a normal distribution, (p value >.05). The normal P-P Plot was used, and it was found that there was a linear relationship between independent and dependent variables. There was no multicollinearity among independent variables. Simple linear regression and forward multiple linear regression were used to determine predictors of caregiver burden scores [36].

## Results

The findings were divided into four parts: demographic characteristics of the caregivers and correlation with caregiver burden categories as shown in Table 1. The level of caregiver burden, depression, quality of life and correlation with caregiver burden are shown in Table 2. Demographic characteristics of the care recipient, ADL levels, IADL scores and correlation with caregiver burden are shown in Table 3. Predictors of caregiver burden results by simple linear regression analysis are shown in Table 4 and predictors of caregiver burden results by forward multiple linear regression analysis are shown in Table 5.

**Table 1:** Demographic characteristics of caregiver and correlation with caregiver burden categories.

Measure	Item	Count (n)	Percentage (%)	Mean	p-Value (Fisher’s)
**Gender**	Male	14	20.3		.457
	Female	55	79.7		
**Age (years)**	18–39	15	21.7	58.46 SD 12.76	.000*
	40–59	20	29.0	SD 12.76	
	60+	34	49.3		
**Marital Status**	Single	6	8.7		.324
	Married	55	79.7		
	Widowed	5	7.2		
	Divorced	3	4.3		
**Family Type**	Nuclear	35	50.7		.200
	Extended	34	49.3		
**Education Level**	Primary or less than	44	63.8		.002*
	Middle and High School	20	29		
	Bachelor or higher	5	7.2		
**Occupation**	No Occupation	15	21.7		.496
	Laborer	22	31.9		
	Trader	11	15.9		
	Farmer	10	14.5		
	Other	11	15.9		
**Older People**	1	56	81.2		.621
**In Home**	2	12	17.4		
	3	1	1.4		
**Relationship Recipient**	Child	34	49.3		.009*
	Niece or Nephew	3	4.3		
	Spouse	24	34.8		
	Sibling	3	4.3		
	Relation Other	5	7.2		
**Income Level**	Less than 3,000 Baht	37	53.6		.041*
	3,001–9,000 Baht	17	24.6		
	More than 9,001 Baht	15	21.7		
**Hours of Care**	0 to 40	37	53.6	52.42	.021*
**Per Week**	41 to 80	8	11.5	SD 40.16	
	More than 81 Hours	24	34.8		

**Table 2:** Level of caregiver burden, depression, quality of life and correlation with caregiver burden.

Measure	Item	Count (n)	Percentage (%)	Mean	p-Value (Fisher’s)
**CG Burden**	No Burden	13	18.8		
	Low-Moderate Burden	30	43.5		
	Moderate-High Burden	21	30.4		
	High Burden‎	5	7.2		
Depression (PHQ)*	Depressed	5	7.2		.411
	Not Depressed	64	92.8		
**Quality of Life**	Low QOL	0	0		.001*
	Moderate QOL	43	62.3		
	Good QOL	26	37.7		

**Table 3:** Demographic characteristic of care recipient, ADL levels, IADL scores and correlation with caregiver burden.

Measure	Item	Count (n)	Percentage (%)	Mean	p-Value (Fisher’s)
**Gender**	Male	35	50.7		.269
	Female	34	49.3		
**Age (years)**	60–69	17	24.6	77.26	.422
	70–79	20	29.0	SD 9.68	
	80–89	24	34.8		
	90+	8	11.6		
**ADL Status**	Bedbound	10	14.5	12.74	.003*
	Homebound	18	26.1	SD 6.26	
	Social Bound	41	59.4		
**IADL Scores**	0	23	33.3	2.57	.258
	1	11	15.9	SD 2.67	
	2	7	10.1		
	3	5	7.2		
	4	4	5.8		
	5	5	7.2		
	6	5	7.2		
	7	6	8.7		
	8	3	4.3		

**Table 4:** Predictors of caregiver burden results of simple linear regression analysis.

**Variable**	**B**	**β**	**t**	**95% CI**	**p**
**ADL Total**	-.850	-.545	-5.326	-1.168, -.531	.000
**IADL Total**	-1.745	-.478	-4.458	-2.524, -.964	.000
**QOL Total**	-.442	-.564	-5.596	-.600, -.284	.000
**PHQ Total**	1.654	.601	6.148	1.117, 2.191	.000
**Caregiver Age**	.305	.399	.399	.134, .476	.001
**Care Recipient Age**	.092	.091	.750	-.153, .336	.456
**Hours of care/week**	.103	.424	3.835	.049, .157	.000

**Table 5:** Predictors of caregiver burden results of forward multiple linear regression analysis.

Variable	B	β	t	p
**(Constant)**	24.732		8.270	< .000
**ADL Total**	-.532	-.341	-3.662	.001
**PHQ Total**	1.495	.543	6.899	< .000
**Hours of care/week**	.055	.227	2.456	.017
R squared = .608	Adjusted R Square = .590	F = 33.663		

### Characteristics of caregivers

Of the 69 caregivers 55 were female (79.7%) with a range of caregivers ages from 19–83 years old. The mean age was 58.46 ± 12.76: 55 (79.7%) were married, 34 (49.3%) were the child of the care recipient and 24 (34.8%) were the spouse of the care recipient. Most of the caregivers (44, 63.8%) had a primary school or below education. A majority (53.6%) of caregivers reported earning less than 3,000 baht (approx. 90 USD) per month with 37 (53.6%) providing 40 hours or less of care per weeks. Fisher’s Exact Test was used to find the association between categorical caregiver burden to caregiver factors. The significant factors (p < .05) were: age of the caregiver (p = .000), education level of the caregiver (p = .002), relationship to the care recipient (p = .009), categorical income level of the caregiver (p = .041), and categorical levels of hours of caregiving per week of work (p = .021) (Table 1).

Of the 69 caregivers assessed, 13 (18.8%) reported no caregiver burden, 30 (43.5%) low-moderate, 21 (30.4%), moderate-high and 5 (7.2%) high burden. Five (7.2%) fit the criteria for being depressed. No caregivers reported a low QOL, 43(62.3%) reported a moderate QOL, and 26 (37.7%) reported a good QOL. QOL of the caregiver (p = .001) was found to be a significant factor (Table 2).

### Characteristics of care recipients

Of the care recipients 35 (50.7%), were male and 34 (49.3%) were female, the average age of the care recipients was 77.26 ± 9.68 with a range of 60 to 99 years old. The caregivers reported that care recipients had the following physical disabilities; 18 (26%) weak legs, 14 (20%) osteoporosis, 11 (16%) stroke, 10 (15%) arthritis, 8 (12%) bone fractures, 5 (7%) kyphosis, and 3 (4%) knees bowing. Using the Barthel Index, the ADL status of the care recipients is as follows; 10 (14.5%) were bed bound, 18 (26.1%) homebound, and 41 (59.4%) social bound. Twenty-three (33.3%) care recipients scored 0 for Lawton-Brody IADL Scale meaning they participated in none of the assessed IADLS. Using Fisher’s Exact Test, of the care recipient factors only the ADL status of care recipient (p = .003) was found to be significantly correlated with caregiver burden (Table 3).

### Predictors of caregiver burden

Numerous factors were significantly associated with caregiver burden total scores when using simple linear regression. Total scores were used of the different variables as they were in a continuous format, this allowed the analysis to be performed. The results were ADL score (p < .000), IADL score (p < .000), QOL score (p < .000), PHQ score (p < .000), caregiver age (p = .001) and hours of care/week (p < .000) were all found to be associated with caregiver burden total scores (p < .05) (Table 3). The significant factors with the greatest changes to caregiver burden total scores were IADL score (B = -1.745) and PHQ score (B = 1.654) (Table 4).

A forward stepwise multiple linear regression analysis was performed to determine predictors of the caregiver burden total scores using modifiable factors that were significant in the simple linear regression analysis. ADL score, IADL score, PHQ score, QOL score, and hours of care/week were entered. All except QOL and IADL were significant (p>.05) (Table 4). A forward multiple linear regression analysis resulted in a significant relationship (R2 adjusted = .590) with 59% of the total variance explained by these three factors. The significance of these factors in descending order are: PHQ score (β = .543, p < .000), ADL score (β = -.341, p = .001), and hours of care/week (β = .227, p = .017) (Table 5). The burden of the caregivers in this study was found to be higher in those who performed more hours of caregiving per week, cared for less independent family members and had higher PHQ score scores (Table 5).

## Discussion

Thailand caregivers will continue to be challenged as the country’s population of people over 60 grows, reaching the super-aged definition by 2030 [3, 4]. In addition, fewer children and urbanization will leave those that care for older people with less family support. These factors make it important to better understand predictors of caregiver burden in caregivers of older people, especially in rural areas where organized support is less readily accessed [7].

When looking at caregiver burden, more than half of the caregivers (62.3%) reported no (18.8%) and low-moderate (43.5%) caregiver burden, this leaves 37.6% of caregivers reporting moderate-high (30.4%) to high care (7.2%). An explanation for the reported levels of caregiver burden could be because of the significant demographic characteristics of the caregivers. Nearly half (49.3%) of the caregivers were found to be over sixty years old; and age was significantly associated with caregiver burden. This might be due to the physical aspects of caregiving and the decline in physical strength and stamina that comes with increasing age. The related factors of education and income were shown to be significantly associated with caregiver burden. The income of more than half (53.6%) of the caregivers is less than 3,000 baht (approx. 90 USD) a month and they have less than a primary school education (63.8%). Being a caregiver can be expensive as they have to pay for medical appointments and medical equipment while having reduced time to work [18]. A study that reported older people in Thailand especially in rural areas still lack access to medical equipment and products for caring or devices due to their high price [37]. A study also found that the caregiving burden of unemployed caregivers was higher than that of their employed counterparts, whether that was due to financial aspects or time spent away from care recipients, both or neither was not determined [19]. The level of burden was not associated with the gender of the caregiver (p = .547) and there were substantially more female caregivers (79.7%) than male. Both of these findings are consistent with other studies that found most caregivers are female and the sex does not impact caregiver burden levels [19, 38, 39]. Five caregivers were categorized as being depressed, without a significant correlation with high caregiver burden levels. More than half of the caregivers (62.3%) were identified as having moderate QOL, with the remaining (37.7%) having high QOL. No one was assessed for having low QOL. Having moderate QOL was significantly correlated (p = .001) with having higher levels of caregiver burden. A Japanese study looking at caregiver burden for family members caring for patients after strokes found some similar results, increased caregiver burden being significantly associated to lower QOL and mental health [40]. Looking at caregiver demographic information, there are factors that are not easily modifiable or not modifiable at all that are significantly correlated. They are, the age of the caregiver (p = .000), education level of the caregiver (p = .002), relationship to the care recipient (p = .009) and categorical income level of the caregiver (p = .041).

There are identified predicting factors that can be analyzed with caregiver burden total scores which give an insight into their relationship and the strength of that relationship. The most significant predictor associated with caregiver burden totals were ADL, depression and hours of care per week. ADL independence level of the care recipient (β = -.341, p = .001), hours per week of work provided by the caregiver (β = .227, p = .017, and depression levels (β = .543, p < .000), of the caregiver were responsible for nearly 60% of the variance in caregiver burden total scores. A Thai study found conflicting results to ours when looking at caregiver burden. They reported that caregiving hours did not significantly affect the well-being of caregivers. They attributed caregiving hours as not being significant due to close relationships creating feelings of generosity and good wishes [41]. This is in contrast with the present study and previous studies completed [17, 26]. They found that the functional ability of care recipients did significantly affect the well-being of caregivers, which is in alignment with the results from this study [41]. The impact of functional ability of the care recipient on caregiver burden is confirmed by two more studies looking at caregivers of patients with strokes, which saw that as the patient’s functional independence changed, so did the caregiver burden [42, 43]. These adjustable factors give an insight into areas on which to focus to improve the caregiver burden.

Many countries are facing challenges related to caring for an aging and disabled population [11, 12]. By better understanding the current challenges, families and government can better support current caregivers and plan for the continually increasing needs of caregivers in the future [28]. With greater caregiver support it will improve the quality of caregiving to better support older people with disabilities. By researching caregiver burden, depression, quality of life of caregivers and how they interact, we can gain a better understanding of the experiences of caregivers [44].

The data from this research suggest areas for interventions and future studies. They should focus on increasing independence of care recipients, fewer hours of work required by the caregivers, and reducing depression levels of caregivers. To address the independence of care recipients, healthcare professionals such as occupational therapists and physical therapists could be consulted. The introduction and use of equipment, education and training of family and care recipients, or physically strengthening the care recipient may improve independence. A study supported the view that coordination between organizations in the area which include local government or the municipal office, local hospital, public health staff and care volunteers needs to be done effectively in order to support older people and their caregivers [45, 46].

With greater independence of the care recipient, it is expected fewer hours would be required of the caregiver. Another option to reduce the workload of caregivers is through advocating the sharing of workload with other family members or hiring a paid caregiver for respite-like care. Delegating tasks to other family members would allow the primary caregiver to complete their own chores, leisure activities, work or rest. A hired caregiver could achieve this same purpose, but the financial aspect would be a barrier to many of the assessed participants. The results of significant correlation of income and caregiver burden (p = .041) is not unique to this study, as studies looking at unemployed versus employed caregivers also showed the same correlation [19, 20]. Depression has an influence on the psychological domain and causes difficulty in carrying out daily routines due to fatigue and loss of motivation. A previous study found that it significantly brings down the quality of care. Therefore, it is important to find solutions to factors impacting depression in order to improve quality of care [47]. An intervention of psychotherapy sessions could be conducted to see if that would reduce scores of depression levels and thus associated caregiver burden. Another area for future study would be to interview caregivers to see their suggestions for ways that they could be helped. The interviews would present the researchers with actionable areas to support caregivers that the caregivers themselves perceive as most beneficial. Continued further research is needed to better prepare families, government, and society to care for aging populations. These results can be used elsewhere to help build a framework for providing support. Countries of more similar culture and economic conditions can more easily apply the research.

There were several limitations noted with this study. All the data was reported by caregivers, this is inherent with the assessment methods used. Caregivers also answered all questions in place of care recipients who were not interviewed due to COVID concerns and their varied cognitive status. Cognitive assessments were not carried out, this study only focused on physical impairments and related caregiver burden. Future studies could incorporate the physical and cognitive status of the care recipients. The influence of over helping may play a part in shifting the scores lower for care recipient’s ADLS and even more IADLs levels. Children, especially, may not want their parents to go out shopping or to do laundry. A post hoc analysis was completed after data collection. A sample power was run for multiple regression (α = 0.05, medium effect size, predictors = 3, participants = 69) resulting in a power of 0.75 which is close to 0.80. The results from this research are valuable information for other researchers to study. Further research could expand into more areas to increase the sample size for stronger statistical power to be more generalized in other communities.

## Conclusion

This study found age, education level, income level, and hours of care directly correlated to caregiver burden level. Most caregivers had a low-moderate level of burden and moderate quality of life. Quality of life was a significant factor associated with caregiver burden level. When categorized, only the ADL level of the care recipient was associated with caregiver burden. When looking at total scores, ADL, IADL, QOL, depression, caregiver age, and hours of care provided per week were all found to be predictors of caregiver burden. When taking the previous factors all considered together, ADL of the care recipient, depression score of the caregiver, and total hours of care provided were three significant predictors. To be effective in reducing the burden of the caregivers, modifiable factors such as training ADL for older adults with disabilities and providing education on caring for the caregivers should be the main focus. In addition, developing a community center for rehabilitation and day care services can reduce hours of care for the caregivers.
